# Hepatic regenerative response to long-term consumption of cinnamon-rich diet in aged rats

**DOI:** 10.22038/AJP.2023.21751

**Published:** 2023

**Authors:** Mahmoud Zardast, Samaneh Nakhaee, Mohammad Attarzadeh Firouzabadi, Mohammad Dastjerdi, Masumeh Askari, Zahra Ghiravani, Khadijeh Farrokhfall

**Affiliations:** 1 *Cardiovascular Diseases Research Center, Birjand University of Medical Sciences, Birjand, Iran*; 2 *Medical Toxicology and Drug Abuse* *Research Center, Birjand University of Medical Sciences, Birjand, Iran*; 3 *Department of Surgery, School of Medicine, Zahedan University of Medical Sciences, Zahedan, Iran*

**Keywords:** Cinnamomum Zeylanicum, Liver resection, Oxidative stress, Ageing, HGF, TNF-α, Nitric oxide, Regeneration

## Abstract

**Objective:**

The present study aimed to investigate the impact of cinnamon on liver regeneration in a rat model of partial hepatectomy (PH).

**Materials and Methods:**

Thirty-two old male Sprague-Dawley rats (12 weeks old) were randomly divided into two equal groups (n=16). One group was fed with a standard diet (control) while the other group was fed with the same diet containing 1% cinnamon for 41 weeks. Then, all animals were subjected to the PH procedure and their livers were studied on postoperative days 2, 10 and 28. The liver contents of hepatic growth factor (HGF), insulin, malondialdehyde (MDA), nitric oxide metabolites (NOx), superoxide dismutase (SOD) and tumor necrosis factor-alpha (TNF-α) were evaluated. Also, the serum levels of liver function markers (alanine aminotransferase (ALT) and aspartate aminotransferase (AST), MDA, NOx and SOD activity were measured.

**Results:**

The regenerated liver weight was significantly higher in cinnamon-treated animals than the controls on both day 10 and 28 post hepatectomy. The hepatic MDA levels in the cinnamon-treated animals were significantly lower than the control rats. Cinnamon led to a significant increase of SOD on day 2 after hepatectomy in serum and liver content. The basal level of HGF in the liver of cinnamon-consuming rats was significantly higher than in the control rats. Hepatic insulin level was significantly increased relative to baseline and control on day 2 in the cinnamon-consuming rats. Hepatic TNF-α levels dramatically decreased on postoperative days (POD) 2 relative to baseline in the control and cinnamon-treated rats.

**Conclusion:**

Long-term cinnamon consumption enhanced liver regeneration outcomes in old rats.

## Introduction

Senescence is associated with loss of activity in homeostasis maintenance due to structural or functional disturbances, consequently making hepatocytes prone to external trauma and damage. The world's elderly has been increasing steadily (Frith et al., 2009). Aging is associated with a gradual alteration of hepatic structure and function and poor prognosis of common non-infectious liver diseases, including nonalcoholic/alcoholic fatty liver diseases (Kim et al., 2015). On the other hand, liver cancers also increase with senescence. Liver regeneration is needed to protect against all above-mentioned liver diseases. The unique capability of the liver to overcome diverse and long-term liver injury is achieved by the auto-proliferative activity. Healthy liver can recover the lost mass or injury through regeneration. Numerous growth factors/cytokines and multiple pathways are involved in different steps (priming, proliferation, and termination) of liver regeneration, including tumor necrosis factor-a (TNF-α), hepatocyte growth factor (HGF), and insulin (Tao et al., 2017). 

Insulin and HGF are the proliferation factors that promote the second phase of liver regeneration. Activation of HGF is crucial for liver regeneration. HGF, as a complete mitogen, provides an early signal for hepatocyte proliferation. Insulin is expressed as an axillary mitogen involved in liver regeneration (Michalopoulos, 2010). 

It is now recognized that nitric oxide (NO) plays an important role in liver regeneration. NO is essential for the early regeneration phase and is induced by several genes involving liver regeneration, such as nuclear factor-kB (NF-kB). Also, NO acts as a regeneration controller due to its effects on apoptosis/proliferative balance. Therefore, any disturbances in NO have detrimental effects on the regeneration process (Carnovale and Ronco, 2012; Helling, 2006).

Other factors that contribute to liver regeneration are reactive oxygen species (ROS). Oxidative stress and ROS production are increased in both liver resection and aging (Enkhbold et al., 2015; Pibiri, 2018). Oxidative stress in the liver remnant may impose a crucial role as a negative regulator in the regeneration process. Hepatic cytochrome P450 and protective enzyme superoxide dismutase (SOD) activity are diminished with increasing age, which may contribute to increased sensitivity of hepatocytes to external substances (Frith et al., 2009). 

 The recovery of patients after liver resection depends on the regenerative capability of the remnant liver. If liver regeneration is impaired, liver failure may occur. Accordingly, a new therapeutic strategy is needed for protection against liver injury and improvement of regenerative capacity. Natural products are a healthy and attractive recommendation in this circumstance, especially for the aged population. Cinnamon (*Cinnamon zeylanicum* or *true/ Cylane Cinnamon*) is a traditional spice widely used worldwide as a food additive (Haidari et al., 2020). Previous studies have shown that its mechanism of hepatoprotection is related to its antioxidant and anti-inflammatory effect, inhibition of apoptosis, etc. (Eidi et al., 2012; Moselhy and Ali, 2009). The beneficial effect of cinnamon is attributed to the main constituent essential oil of cinnamon, cinnamaldehyde (Ataie et al., 2019; Suryanti et al., 2018). Also, a recent study has proved the valuable effect of cinnamon on oxidative stress and postoperative recovery of liver resection in aged animals (Dastjerdi et al., 2020). However, the beneficial/deleterious effect of cinnamon on liver regeneration has not been studied. 

In this study, the effects of cinnamon consumption on liver regeneration rate and several regeneration mediators in an aged rat model of liver resection have been investigated. The accurate determination of therapeutic effects and longitudinal assessment of liver regeneration molecules are critical to identify long-term impaired/improved regeneration. For this purpose, assessments at different time points after liver resection may provide more information on liver regeneration in preclinical research. 

## Materials and Methods

**Animals and study design**

Male Sprague Dawley rats (32 rats, 12 weeks old) were supplied from the Research Centre of Experimental Medicine of Birjand University of Medical Sciences (BUMS). All experiments were conducted in accordance with the relevant guidelines and regulations, and the local ethics committee of Birjand University of Medical Sciences approved the study (IR.BUMS.REC.1397.195). The study is reported in accordance with ARRIVE guidelines (Lu et al., 2009). Animals were kept in appropriate laboratory conditions with a 12-hr light/ dark cycle and 24±2^°^C temperature, and they had free access to water and food. Rats were acclimatized to the laboratory condition for one week. 

Then, they were randomly allocated into two equal groups; control and cinnamon. Animals in the control group were treated with the standard laboratory animals’ diet (Javaneh Khorasan Company, Iran) whereas, the cinnamon group received the standard diet supplemented with 1% (w/w) of cinnamon powder (Javaneh Khorasan Company, Iran) for 41 weeks. Based on the daily food consumption of rats (Reagan-Shaw et al., 2008) and the maximum allowed dose of this spice in humans (Khan et al., 2003), the concentration (1%) was chosen. During the study period, animals’ weight and food intake were monitored weekly. Four rats from each group were randomly selected as sham-operated control animals. The sham animals had surgery without liver resection; instead, the rats were subjected to midline laparotomy, liver manipulation, and analgesic medication (pentobarbital, 60 mg/kg, intraperitoneal (IP). They were sacrificed 24 hr later, and their blood/liver samples were collected and considered control. The remaining animals of each group (n=12 each) underwent partial hepatectomy (30% liver weight was removed). In order to evaluate the hepatic regeneration capacity in the studied groups, blood and liver specimens (n=4 each) were collected at different time points following the procedure (48-hr, 10 days and 28 days). Following 12-14 hr fasting, animals were anesthetized using pentobarbital (60 mg/kg, IP) and blood was taken from the heart. Blood was transferred to tubes with no anticoagulant to collect blood serum. Blood samples were centrifuged at 3000 g for 10 min, and then serum was separated and stored at -20°C until biochemical evaluation.

**Hepatectomy procedure**

 Liver resection was carried out in accordance with the Higgins hepatectomy method (Higgins, 1931) with some modifications, as mentioned previously (Dastjerdi et al., 2020). Briefly, after anesthesia with pentobarbital (60 mg /kg, IP), a transverse abdominal incision (1.5-2 cm) was made, and the base of the left lateral (anterior) hepatic lobe was first ligated and then carefully dissected (30% of liver was removed) (Martins et al., 2008). Moreover, the coronary ligaments, as well as the surrounding liver lobes, were conserved and the hepatic tissue was dissected from the site immediately after the ligation. Liver resection was carried out with caution to avoid any damage to the liver tissue. When the resection was accomplished, the remaining parts of the hepatic parenchyma were carefully checked for clear blood and lack of congestion to make sure that ligation did not stand too low and would not cause any partial obstruction in one of the main hepatic veins or even in venae cavae. The abdomen incision was closed using a two-layer closure with stitches of 0.4 nylon sutures. Immediately after the recovery, rats received an oral dose of ibuprofen (30 mg/kg) and then transferred to a transparent cage. The rats were kept under a heating lamp until complete recovery from anesthesia. Surgery was carried out by skillful personnel at 9-12 AM under aseptic conditions. All hepatectomy procedures were done during 20-25 min.

**Measurement of liver regeneration rate**

The resected portion of the liver and the regenerated liver after 2, 10, and 28 days following hepatectomy were weighed using an electronic balance (0.001 g). The liver regeneration rate (%) was calculated as the percentage of preoperative estimated liver weight per 100 g body weight to liver weight at sacrifice per 100 g body weight (Kwon et al., 1990).

**Tissue preparation**

Liver samples were washed and homogenized in cold PBS (phosphate buffered saline, pH 7.4). The homogenate mixture was centrifuged (10000 rpm, 20 min, at 4°C), and the supernatant was collected for the determination of lipid peroxidation (MDA), nitric oxide metabolites (nitrate + nitrite= NOx), superoxide dismutase (SOD), insulin, TNF-α, and hepatocyte growth factor (HGF) as follows.

**Biochemical measurement**

**Liver NOx measurements**

NOx contents in the liver samples were measured by the Griess reaction as previously described (Farrokhfall et al., 2015). In brief, the liver supernatant samples were deproteinized by ethanol (equal volume to the sample volume), shacked vigorously, and centrifuged at 10000 rpm for 10 min and supernatants were collected. NOx was measured by the reduction of nitrate to nitrite with saturated vanadium chloride solution (0.8% in HCl solution 1 M). Then, the Griess reagent for assay reaction and color formation (sulfanilamide (2% in 5% HCl solution) and N-(1-naphthyl) ethylenediamine dihydrochloride (NEDD, 0.1% in distilled water) were added. The incubation was done at 37°C for 30 min. The colored complex was read at 540 nm wavelength. NOx concentration was calculated from the standard curve constructed with 0-80 μmol sodium nitrate. 

**Measurement of serum and liver MDA**

The levels of thiobarbituric acid–reactive substance, as a byproduct of lipid peroxidation, were determined in serum and liver extract based on the reaction with thiobarbituric acid (0.67%) in acidic pH at 90–100°C. The absorbance of the resulting pink product was measured spectrophotometrically at 535 nm and MDA concentration was expressed as μmol/L (Nakhaee et al., 2021).

**Other biochemical assays**

Liver insulin (Mercodia, Sweden), HGF (Zelbio, Germany) and TNF-α (Diacolon, France) were measured using rat specific ELISA kits. Serum alanine aminotransferase (ALT) and aspartate aminotransferase (AST) levels were measured using the Modular P (Roche Diagnostics, Mannheim, Germany). Liver total SOD activity was determined using a commercial kit (Nasdox™–Superoxide Dismutase Non-Enzymatic kit; Code: NS-15033, Navandsalamat, Iran). Finally, the enzyme activity was calculated as (U/ml) =OD test/OD control ×200. 

**Data analysis**

Data were analyzed by nonparametric Kruskal-Wallis test and then "Dunn's Multiple Comparison Test" was used for two groups’ comparison. Data of body weight and food intake was evaluated by two-way repeated measure ANOVA (RM ANOVA) followed by Bonferroni post-tests. In all the tests, p<0.05 was considered significant. 

## Results

The results of sham groups (Animals that had the all condition of hepatectomy surgery - midline laparotomy, liver manipulation, and analgesic medication - without liver resection) was explained as control in Figure 3 and 4 and Table 1. 

**Effect on body weight and food intake**

**Figure 1:**
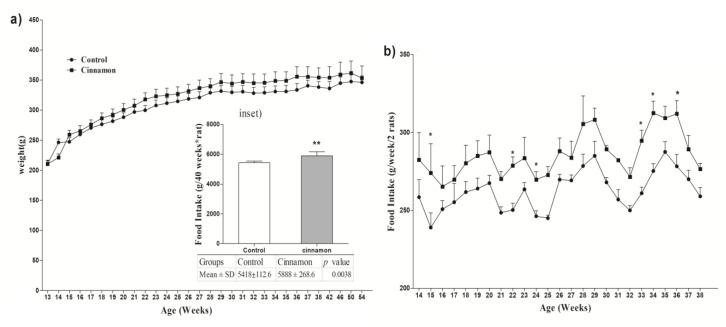
Evaluation of rat body weight (a) and food intake (b) for each experimental group. Cumulative food intake during study is presented in inset. Diet including cinnamon 1% was given for 41 weeks instead of regular diet in the cinnamon group. Values are expressed in mean (g) ± standard error. Data were analyzed by two-way ANOVA followed by Bonferroni post-tests" for comparison of the two groups at each time point. *p<0.05 significant versus the control animals at the same time point.

No significant differences were observed between the two groups following 41 weeks of receiving regular and cinnamon-rich diet (~1% w/w) as shown in Figure 1a. The weight gain (g) in the control and cinnamon consumed rats was 135.6±25.51 and 142.6±42.53, respectively (p=0.7). For evaluation of the cinnamon effect on rat feeding, the food intake was weekly measured. The data showed that the cinnamon-treated rats ate significantly more than regularly-fed rats (Figure 1b). 

**Effects on liver index (%liver /body weight)**

**Figure 2:**
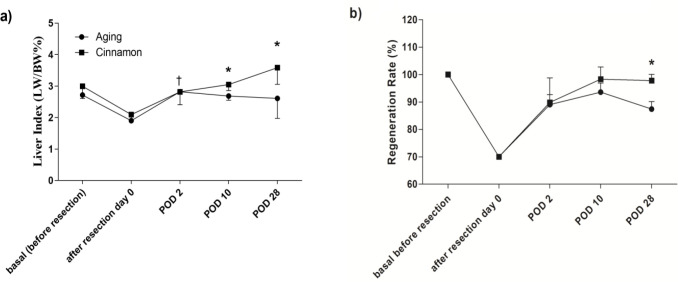
Regenerated liver weight index (a) and liver regeneration rate (b) after liver resection for each experimental group. Data are expressed based on median and error [interquartile range] of four animals in each group except for control (n=12). Data were analyzed by Kruskal-Wallis test followed by Dunn's Multiple Comparison Test in each treatment and Mann-Whitney test in comparison of each point in different treatment. *p<0.05 versus aging animals in same post-operative fallow up day; †p<0.05 versus estimated liver weight before resection in same treatment LW/BW: liver weight/ body weight.

Long-term cinnamon feeding did not alter the liver index (median±IQR; 2.71 [2.60; 3.02] and 2.99 [2.73; 3.21, p=0.45] in the control and cinnamon groups, respectively). However, the regenerated liver index was significantly higher in the cinnamon-treated animals than the controls on both 10 and 28 post hepatectomy days (Figure 2a). The liver index gradually increased in the cinnamon treated rats as the liver index was completely restored 28 days after liver resection (Figure 2a). Nevertheless, we did not find this pattern in the control animals (Figure 2a). 

To evaluate liver regeneration rate after liver resection, the liver regenerated ratio (RR) was calculated in both groups (Figure 2b). The RR exhibited a pattern similar to liver weight. The RR of the cinnamon group was significantly higher than the control group on both 10 and 28 post hepatectomy days.

**Effects on oxidative stress markers**

**Figure 3:**
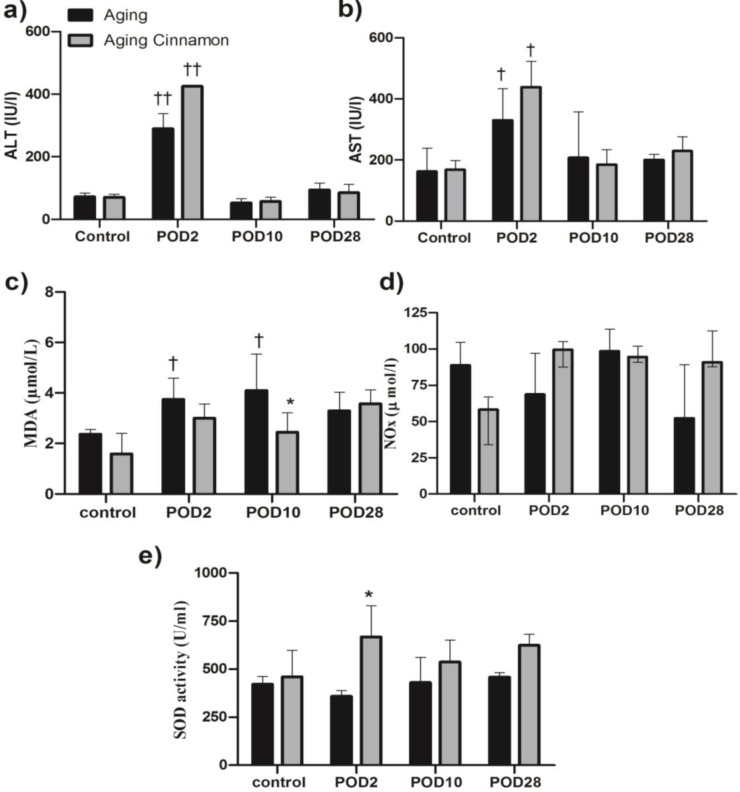
Effect of cinnamon and hepatectomy on liver oxidative/ nitrosative stress Results are expressed based on median [interquartile range] of four old animals in each group. Diet rich cinnamon 1% was fed 41 weeks instead of regular diet in aging cinnamon. Data were analyzed by Kruskal-Wallis test followed by Dunn's Multiple Comparison Test. *p<0.05 versus aging group in same post-operative fallow up day†p<0.05, ††p<0.01 versus control in same treatment. ALT: alanine aminotransferase, AST: aspartate aminotransferase, MDA: malondialdehyde, NOx: nitric oxide metabolites, SOD: superoxide dismutase, POD: post-operative day.

**Figure 4:**
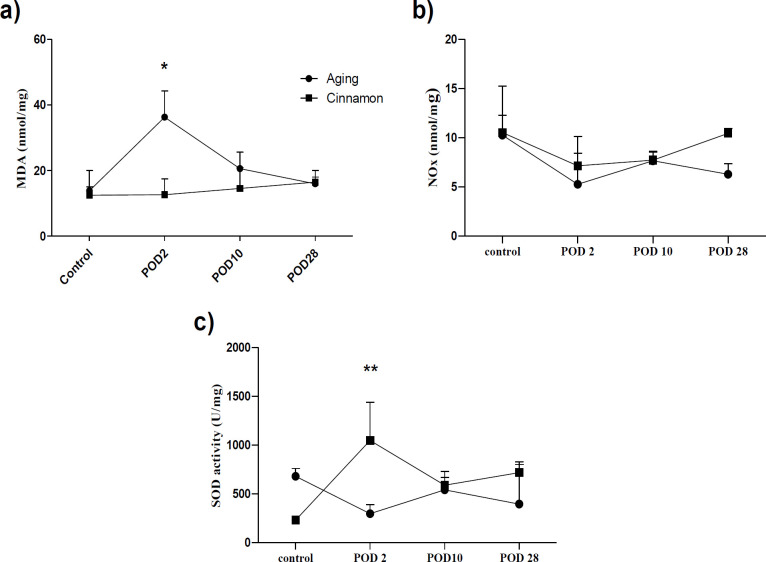
Effect of cinnamon and hepatectomy on liver oxidative/ nitrosative stress.Results are expressed based on median [interquartile range] of four old animals in each group. Diet rich cinnamon 1% was fed 41 weeks instead of regular diet in aging cinnamon. Data were analyzed by Kruskal-Wallis test followed by Dunn's Multiple Comparison Test. *p<0.05, **p<0.01 versus aging group in same post-operative fallow up day. MDA: malondialdehyde, NOx: nitric oxide metabolites, SOD: superoxide dismutase, POD: post-operative day.

**Figure 5:**
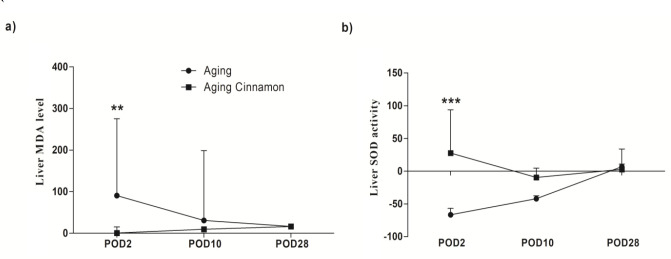
Change in Lipid peroxidation (a) and SOD activity (b) level relation to before resection (%) in liver. Values are median and error [interquartile range] of four animals in each group. Data were analyzed by Kruskal-Wallis test followed by Dunn's Multiple Comparison Test in each treatment and Mann-Whitney test in comparison of each point in different treatment. **p<0.01 high significant versus aging animals in same post-operative fallow up day; *** p<0.001 very high significant versus aging animals in same time point. POD 2: post-operative day 2, POD10: post-operative day 10, POD28: post-operative day 28, MDA: malondialdehyde, SOD: superoxide dismutase.

We examined MDA and NOx as oxidative/nitrosative stress markers and SOD for antioxidant power in the experiment. At the 48-hr post hepatectomy, the cinnamon-treated animals exhibited significantly higher serum/ liver SOD activity than their counterparts in the control (Figure 3 and 4). In the cinnamon group, the liver SOD activity was dramatically increased 48-hr post hepatectomy compared to the control; however, the enzyme activity was no-significant decrease on the POD 2 in control group (Figure 4c). Lipid peroxidation significantly increased 48 hr after the liver resection in the control group (p<0.05; Figure 4a). In addition, for elucidation of liver oxidative system fluctuations, the markers were assayed in the resected liver lobe and in the recovered liver in both groups then the percent changes were calculated and presented (Figure 5). The liver MDA was significantly elevated on postoperative day (POD 2) compared with cinnamon at each time point, namely, cinnamon prevented liver MDA rise (i.e. the percent of liver MDA change 48 hr after liver resection was 90.58% [50.93; 275.2] and -0.19% [-9.35; 14.9] in control and cinnamon treated animals, respectively; Figure. 5a). The variation disappeared on 28 days after surgery i.e., (16.40% [14.35; 19.05] in control versus 33.23 [-10.81; 71.56] in cinnamon rats; Figure. 5a). In contrast, the ratio of change SOD activity relation to control was significantly higher on POD2 of cinnamon-treated rats compared with control animals at this time point (i.e. SOD activity ratio on POD2 in the cinnamon group increased by 27.51 [19.71; 93.77] whereas it decreased to -66.59 [-81.73; -56.81] in control rats of the same as time point; Figure. 5b). This pattern was lost over time as it had no difference on POD10 in both treatments (Figure. 5b). 

No significant difference was found in the liver or serum NOx between the experimental groups (Figure 3d and 4b). 

**Serum aminotransferases increased after liver resection.**

ALT and AST in the control rats were significantly elevated 48 hr after liver resection compared with the control animals. Long-term cinnamon consumption had no effect on the liver, as no significant differences were found between the groups at different time point. Following liver resection, serum levels of ALT and AST were increased and it was associated with elevated in hepatocytes demise and liver inflammation with the same pattern both in the control and cinnamon groups on POD2 (Figure 3a and 3b). 

`

**Effects on HGF, Insulin, and TNF-α**

Liver resection resulted in slow enhancement of liver HGF as it markedly increased POD28 in control animals (p<0.05, Table 1). Cinnamon consumption for almost 41 weeks dramatically increased the basal level of liver HGF compared with the control animals (p<0.05, Table 1). 

**Figure 6:**
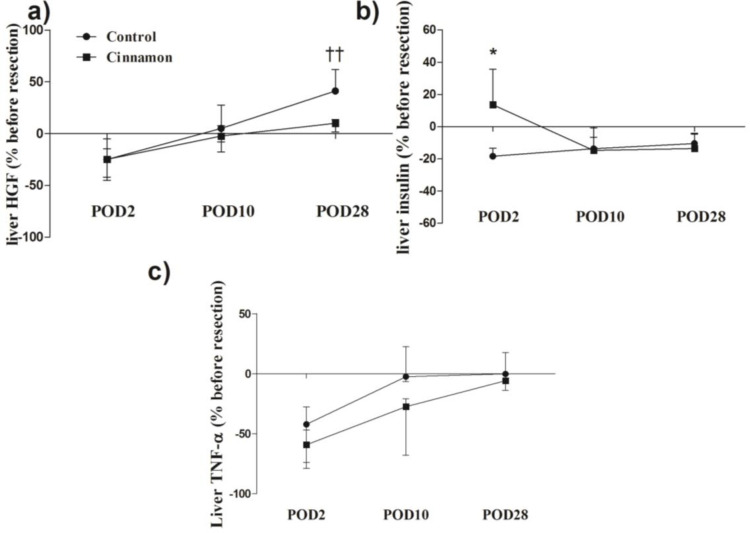
Variation in liver HGF (a), insulin (b) and TNF-α (c) levels relation to before resection (%). Values are median and error [interquartile range] of four animals in each group. Data were analyzed by Kruskal-Wallis test followed by Dunn's Multiple Comparison Test in three time point of each treatment and Mann-Whitney test in comparison of each point in different treatment. *p<0.05 significant versus aging animals in same time point; †† p<0.01 very significant versus POD2. POD 2: post-operative day 2, POD10: post-operative day 10, POD28: post-operative day 28, TNF-α: tumor necrosis factor-alpha, HGF: hepatocyte growth factor.

The percentage change of liver HGF at different time points increased from POD2 until POD28 in both groups; however, it increased more in control animals than in cinnamon treatment animals i.e. liver HGF increased by 41.07% in the control animals (p<0.05, Figure 6a) and 9% in cinnamon-treated rats on POD28. 

Hepatic insulin level was significantly increased relative to the baseline and control on the POD2 in cinnamon-consuming rats (Table 1). Also, it decreased in control animals on POD2 relative to before resection as shown in Figure 6b. The reduction of liver insulin level 48 hr after liver resection in control animals was 14%. In contrast, it increased 13% in cinnamon rats at this time (p<0.05, Figure. 6b). The liver level of TNF-α significantly decreased on POD2 in both aged and cinnamon groups compared with their related controls (p<0.05, Table 1). This condition was observed in the case of the percentage ratio to before resection in TNF-α, which subsequently returned the baseline on POD28 in control and cinnamon groups (Figure. 6c).

**Table 1:** Effect of cinnamon and liver resection on growth and cytokines factors in experimental groups

	** Liver parameters**			
		**HGF (ηg/l)**	**Insulin (Pmol/l)**	**TNF-α (ηg/l)**
**Aging ** **(median [IQR])**	**Control**	65.32 [58.57; 67.34]	49.24 [43.85; 59.51]	1084 [793.9; 1183]
	**POD2**	71.48 [66.37; 103.6]	43.76 [40.41; 49.20]	696.2 [295.5; 889.3]** †**
	**POD10**	70.42 [62.7; 77.14]	48.11 [40.89; 66.29]	893.4 [647.6; 1149]
	**POD28**	91.74 [73.08; 110.7] †	48.11 [41.72; 59.16]	768.9 [675.9; 1044]
**Cinnamon ** **(median [IQR])**	**Control**	78.96 [72.62; 93.20] *	47.15 [42.72; 57.07]	949.1 [706.8; 1029]
	**POD2**	80.59 [67.53; 94.92]	64.64 [53.59; 69.30] †*	456.2 [196.7; 643.3]** †**
	**POD10**	83.56 [80.42; 99.6]	44.37 [37.02; 60.33]	795.6 [357.8; 979.3]
	**POD28**	77.79 [69.28; 92.97]	49.16 [42.76; 52.55]	923.5 [706.6; 1047]

Results are expressed based on median [interquartile range] of four rats (53 weeks old) in each group. Diet rich cinnamon 1% was fed for 41 weeks instead of regular diet in cinnamon. Data were analyzed by Kruskal-Wallis test followed by Dunn's Multiple Comparison Test. *p<0.05 versus aging animals on the same post-operative fallow-up day; **†**p<0.05 versus control in same treatment.

## Discussion

The present study elucidated that long-term cinnamon consumption (1% w/w for 41 weeks) improved liver resection surgery outcomes. This beneficial effect of cinnamon on the liver is partly attributed to high antioxidant activity and, to a greater extent, to more HGF content in the liver as well as the elevation of liver insulin levels following liver resection.

Liver regeneration in the cinnamon group was different from the control rats 28 days after liver resection surgery. In more detail, the regeneration rate and liver index of rats with a regular diet recovered for 2 days post-operative and then sustained unchanged (Figure 2b). However, it continuously increased in the cinnamon diet until the 28th day. Liver regeneration in the 12-month-old rat has only recovered by 60% to 70% on 7 days after hepatectomy (Beyer et al., 1991; Biondo-Simões Mde et al., 2006). A previous study has shown the liver weight of 1-year-old rats was 91% original liver mass 28th day post-hepatectomy (Beyer et al., 1991).

In present study, the full recovery rate of aged rats was not accomplished and reached to 87% on the 28th day after partial hepatectomy. However, the hepatic mass of cinnamon feeding rats on day 28 was 95%. On the other hand, a study on humans also showed that the liver regeneration at 14 days or 6 months after liver resection was similar in aged patients. Nevertheless, regeneration rate and liver volume were significantly higher after 6 months compared with 14 days post hepatectomy in young people (Zhu et al., 2014). According to the data, the liver of old animals consuming cinnamon can behave like a young liver as the cinnamon consumption has increased HGF levels in the liver. 

Hepatic growth factor, by maintaining the self-repair of the liver, causes the survival of hepatocytes exposed to any liver damage (Nakamura and Mizuno, 2010) and it is necessary for liver regeneration (Huh et al., 2004). 

It seems that the main beneficial effect of cinnamon on liver regeneration is achieved by enhancement of liver HGF content and insulin in old animals. HGF and its receptor cMet (mesenchymal-epithelial transition factor) is the most important pathway for liver regeneration (Huh et al., 2004). HGF plays key roles in development, tissue regeneration, and cell motility. In the liver, it is essential for the self-repair of the injured liver and hepatic regeneration. HGF is an important growth factor in the simultaneous re-entering of hepatocytes to the cell cycle that in turn promotes DNA synthesis for liver proliferation (Nejak-Bowen et al., 2013). The HGF and cMet expression and its signaling function are lower in old patients (Zhu et al., 2014). Enkhbold et al. (Enkhbold et al., 2015) also have shown that the expression of HGF and cMet was diminished in the old animals during 72 hr following partial hepatectomy. We could not find any studies on the issue of the role of cinnamon in hepatic tissue regeneration. However, the effect of cinnamon and its constituents on other growth factors, such as vascular endothelial growth factor (VEGF) and insulin-like growth factor-1 (IGF-1) have been frequently investigated (Choi et al., 2009; Mona, 2010). Cinnamon cassia and cinnamic acid –an active compound of cinnamon- stimulate angiogenesis *in vivo* (mice) and *in vitro* (Human umbilical vein endothelial cells) through up-regulation of VEGF (Choi et al., 2009). Also, it has been reported that cinnamaldehyde (the main constituent of the essential oil of cinnamon) improved wound healing through augmentation of VEGF in diabetic mice (Yuan et al., 2018). It is considered that this impact of cinnamon is different in each context as it promotes wound healing by diminished VEGF in the acute infectious wound (Daemi et al., 2019) and tumor treatment or prevention (Lu et al., 2009, Zhang et al., 2017).

 It has been reported that HGF mediated the compensatory hypertrophic responses of islets to insulin resistance and promoted insulin secretion (Araújo et al., 2012). It is suggested that the high HGF due to long-term cinnamon consumption in aged rats has provoked insulin secretion. The high insulin level of the liver can, in turn, foster the proliferation in the liver regeneration process (Michalopoulos., 2010). 

A study has shown that hepatectomy influences oxidative state of the liver as well as plasma levels of lipid peroxides and antioxidant substances (Andiran et al., 2003). Our results showed that MDA, a biomarker of free radical-mediated lipid peroxidation, was increased in regenerating liver after PH, which was consistent with previous studies (Sánchez-Hidalgo et al., 2012; Toydemir et al., 2013). It has been found that lipid peroxidation begins to occur in a very early phase in regenerating the liver (Sánchez-Hidalgo et al., 2012). In contrast, other studies have reported that liver regeneration takes place under low oxidative stress (Alexandris et al., 2004) and diminished lipid peroxidation contributes to cell division after partial hepatectomy (Cheeseman et al., 1986; Gorla et al., 2001).

Actually, some discrepancies still exist concerning the role of lipid peroxidation during typical cell proliferation following partial hepatectomy. It seems although lipid peroxidation could be a fundamental mechanism of toxicity on cell membranes, it is also a normal physiological process (Khoshtabiat and Mahdavi, 2015).

In addition, PH impairs the antioxidative system, such as SOD, which can offer protection from cell damage by scavenging superoxide anion radicals beyond the reactive oxygen metabolism cascade. Impaired antioxidant defenses decrease oxidative phosphorylation capabilities to finally impede liver regeneration after PH (Yang et al., 2004). Our results further showed a remarkable protective effect of cinnamon, as documented by restoring SOD activity, and alleviation of lipid peroxidation. Accordingly, a part of the beneficial effect of cinnamon on liver regeneration capacity was probably due to its ability to attenuate liver injury and restore antioxidant enzymes activity.

Due to liver resection, the blood flow rate of the remnant liver is increased and results in high shear stress that promotes NO production. Also, NO was mainly produced from Kupffer cells following liver resection. NO, along with TNF-α, is an inducer of the priming phase of liver regeneration (re-enter of hepatocytes from G0 to G1) and potentiates HGF production, which is essential for self-repair of injured liver and hepatic regeneration. The use of NO synthesis inhibitors has impaired the proliferation in liver regeneration (Carnovale and Ronco, 2012; Yagi et al., 2020). A study has confirmed that the increased hepatic NO is necessary for liver regeneration (Carnovale et al., 2000). In the present study, liver NOx did not change following liver resection. It may attribute to the lack of a follow-up study during the first hours after liver resection. There is evidence showing that NO increased early (6 hr) after hepatectomy and returned to normal levels after 16 hr (Hortelano et al., 1995). The same explanation can also state for TNF-α.

Although the study results appended the data on the hepatoprotective and high regenerative capacity of cinnamon, it suffers from some limitations. Firstly, because of high mortality rate (40%) of aged animals (Enkhbold et al., 2015; Sánchez-Hidalgo, 2012), the formal partial hepatectomy (70%) was not performed. Secondly, we investigated only the paracrine role of HGF, TNF-α, and insulin in liver regeneration. It is important that future research investigate other signals, pathways and possible cinnamon side effects. Thirdly, in this study, one dose of cinnamon was administered through diet. Further studies considering the different doses of cinnamon are needed.

In conclusion, cinnamon showed an advantageous effect on liver regeneration, probably by increasing hepatic levels of HGF, insulin and antioxidant capacity. Cinnamon may be an effective health strategy to keep the liver healthier and enhance regenerative capacity against any possible metabolic liver damage or liver resection with life span.

## Conflicts of interest

The authors have declared that there is no conflict of interest.
